# Periodontal Health, Bite Force and Oral Health‐Related Quality of Life of Obese Older Adults Using Removable Partial Dentures

**DOI:** 10.1111/joor.70122

**Published:** 2025-11-27

**Authors:** Guilherme Fantini Ferreira, Talita Malini Carletti, Lorena Tavares Gama, Thais Marques Simek Vega Gonçalves, Renata Cunha Matheus Rodrigues Garcia

**Affiliations:** ^1^ Department of Prosthodontics and Periodontology Piracicaba Dental School, University of Campinas Piracicaba Brazil; ^2^ Department of Dentistry Federal University of Santa Catarina Florianópolis Brazil

**Keywords:** bite force, obesity, older adult, periodontal health, quality of life, removable prostheses

## Abstract

**Background:**

Obesity is associated with periodontal disease, which compromises chewing by reducing masticatory efficiency. However, little is known about periodontal health and the use of removable prostheses in obese older adults.

**Objective:**

To evaluate the effects of removable partial denture (RPD) insertion on periodontal condition, maximum bite force (MBF), and oral health‐related quality of life (OHRQoL) in obese and normal‐weight older adults.

**Material and Methods:**

Edentulous older adults in the maxilla and partially edentulous in the mandible were assigned to obese (71.9 ± 5.0 years; *n* = 12, 6 women and 6 men) and normal‐weight (69.9 ± 6.7 years; *n* = 6 women and 6 men) groups. Periodontal parameters (probing depth, clinical attachment loss, gingival recession, bleeding on probing) were measured with William's probe, and plaque was assessed with the modified O'Leary index. MBF was recorded with pressure sensors, and OHRQoL was evaluated with the OHIP‐14. Periodontal parameters and MBF were measured before and 3, 6, 9, and 12 months after RPD insertion, while OHRQoL was measured before, and after 6 and 12 months. Data were analysed by repeated‐measures ANOVA with Bonferroni post hoc tests (*α* = 0.05).

**Results:**

Obese individuals showed higher bleeding on probing (*p* = 0.02) and greater MBF (*p* = 0.002) at all time points. MBF increased in both groups after 3 months, while OHRQoL remained lower in obese individuals (*p* = 0.01). Both groups showed significant plaque reduction after RPD insertion (*p* < 0.05).

**Conclusion:**

Obese older adults wearing RPDs exhibit greater periodontal impairment and poorer OHRQoL, despite similar improvements in MBF and plaque reduction. These findings underscore the need for closer periodontal monitoring and targeted oral health strategies for obese older adults receiving RPDs.

**Trial Registration:**

Brazilian Registry of Clinical Trials: ReBEC #U1111‐1228‐7273

## Graphical abstract

Impact of removable partial dentures on periodontal health, bite force, and oral health–related quality of life in obese versus normal‐weight older adults. Obese RPD wearers require closer periodontal management due to increased inflammation and lower OHRQoL.

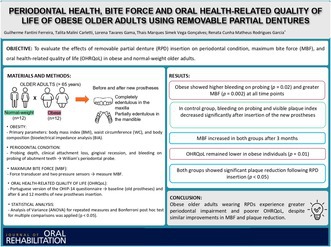

## 1 Introduction

Obesity is a complex multifactorial chronic disease leading to an excessive fat deposition in the adipose tissue [1]. It results from a long‐term energy imbalance in which the energy intake from food is larger than the energy expenditure [2]. Globally, obesity is greater in older adults [3] and it is considered a common metabolic and nutritional disorder [4], associated with comorbidities such as type 2 diabetes, cardiovascular disease, and certain cancers [5, 6, 7, 8].

Besides systemic alterations, obesity has also been linked to oral health conditions [9, 10], such as xerostomia [11] and the presence of caries [9, 10]. It is also known that obesity contributes to an increased incidence and progression of periodontal disease [12]. Clinically, obese individuals have a higher rate of gingival inflammation [13, 14], bleeding on probing [15], and deeper probing depths [16]. Furthermore, Linden et al. [16] also demonstrated that obese older adults have fewer remaining teeth and higher levels of periodontitis, suggesting that obesity is also associated with tooth loss.

A conservative and quick solution to replace those teeth with a good cost–benefit ratio is the insertion of removable partial dentures (RPDs) [17]. These prostheses restore phonetics [18], aesthetics [19], and masticatory function [20, 21], consequently improving patients' quality of life [22]. However, RPD's effects on periodontal health are still controversial. Longitudinal studies have shown that RPDs have been associated with increased gingivitis and periodontitis [23, 24], generating conditions for biofilm formation [25, 26] and increasing the risk of periodontal disease [27, 28], especially in mandibular Kennedy class I [29]. However, a recent systematic review revealed that RPDs do not negatively impact periodontal parameters, especially when regular maintenance is provided [30]. On the other hand, since obesity is associated with higher levels of periodontal diseases, it is reasonable to suppose that obese subjects wearing RPDs may have higher odds for periodontitis development. Despite that, evidence regarding the periodontal health of obese older adults remains limited, especially in RPD wearers.

In general, periodontal disease impairs chewing by reducing masticatory efficiency [31], cross‐sectional area of masticatory muscles [32], and molar bite force [31]. Meanwhile, replacing missing teeth by insertion of RPDs enhances several parameters of masticatory function [21, 33, 34], including maximum bite force (MBF) [35]. According to Vozza et al. [35], obese RPD users had lower MBF compared to those with normal weight. However, they [35] evaluated the MBF of occlusal pairs composed of artificial teeth or composed of artificial and natural teeth, which may influence results. In contrast, Regalo et al. [36] revealed that fully dentate obese individuals have higher MBF than those of normal weight. Given such inconsistencies, it is important to examine further the effects of removable dentures on the MBF of older obese adults. Thus, this study aimed to evaluate the mid‐term effects of removable prostheses insertion in the periodontal condition and MBF of obese and normal‐weight older adults. In addition, considering that MBF and periodontal conditions may ultimately influence the quality of life [31], this study also evaluated the OHRQoL of obese and non‐obese individuals before and after oral rehabilitation with removable dentures. The working null hypothesis of this study is that no differences would be found between obese and normal‐weight individuals after the insertion of removable prostheses in terms of periodontal condition, MBF, and OHRQoL.

## 2 Material and Methods

Periodontal conditions of RPD abutment teeth, MBF, and OHRQoL were the dependent variables of this study. They were evaluated in obese and normal‐weight older adults before and after the rehabilitation with new maxillary complete dentures (CD) and mandibular RPDs, with the weight condition and the follow‐up periods considered independent variables. Older adults were selected based on the criteria explained below. All of them first received general dental treatment and oral hygiene information. The Ethics Committee of Piracicaba Dental School, University of Campinas, approved the research protocol (#10727219.4.0000.5418).

The pre‐rehabilitation assessments (baseline) were conducted with the participants using their old dentures. After, new upper CDs and lower RPDs were inserted, as previously reported [37]. The RPDs frameworks were constructed with a cobalt‐chromium alloy, including T‐shaped clasps, and lingual bars as major connectors. Clinical procedures were conducted by a single dentist with expertise in dental prosthesis, and two technicians performed the laboratory procedures, one responsible for the RPDs frameworks, and another responsible for artificial teeth (Biotone, Dentsply Sirona, York, USA) arrangement and polymerisation of all prostheses.

The periodontal condition of abutment teeth was evaluated in terms of probing depth, clinical attachment loss, gingival recession, bleeding on probing, and visible plaque index. The evaluations were conducted at baseline (with the old prostheses) and following 3, 6, 9, and 12 months of the new dentures' insertion. At each follow‐up visit, the participants received standardised oral hygiene instructions. The MBF was also assessed at the same periods by using a force transducer placed on the artificial first molars region at the same time points [38].

The OHRQoL was measured by applying the Oral Health Index Profile‐14 (OHIP‐14) questionnaire [39], but differently from the previous variables, this analysis was performed at baseline (old prostheses) and after 6 and 12 months of new prostheses use.

### 2.1 Sample

Eligible subjects were aged over 65 years, completely edentulous in the maxilla and partially dentate in the mandible, classified as Kennedy class I, exhibiting only the six anterior teeth (from canine to canine). They should be free of active periodontal disease, show a normal salivary flow rate [40], and wear unsatisfactory prostheses with indications for replacement. All of them were recruited without gender or ethnicity restrictions among those seeking prosthetic treatment in the Dental Clinic of the Piracicaba Dental School, University of Campinas, Brazil. Elders presenting age‐related diseases, such as hypertension and/or diabetes, once controlled by medication were also accepted. Participants with physical impairment, advanced periodontal disease, low salivary flow rate (< 0.6 mg/mL) [40], bruxism and/or temporomandibular disorders history [41, 42] were excluded.

To secure a statistical difference with 80% power and an error probability of 5%, a sample size calculation was carried out based on a previous study [36], considering MBF values of obese and normal‐weight individuals as reference. Additionally, considering periodontal parameters, a sample size calculation was also performed based on probing depth data [16]. The minimum clinically relevant difference was defined as the mean difference between obese and non‐obese participants, corresponding to 232.9 N for MBF [36] and 1.0 mm for probing depth [16]. Data were allocated to the GPower 3.1 software (Dusseldorf, Germany) to calculate the total number of volunteers considering a Comparison of Two Independent Slopes (Two Samples) [43]. Therefore, 10 volunteers per group (obese and normal‐weight) were needed to denote statistical differences. However, 12 volunteers were selected per group, to counterbalance potential losses.

Obesity was assessed using three primary parameters: body mass index (BMI), waist circumference (WC), and body composition, evaluated through bioelectrical impedance analysis (BIA) [44, 45]. BMI was calculated by dividing body weight (kg) by height squared (m^2^) [46]. While BMI is a simple and effective screening tool, it does not differentiate between lean mass and fat mass, potentially leading to an underestimation of obesity [44], particularly in older adults, where changes in body fat distribution are common [45, 47]. Considering this limitation, waist circumference, a key indicator of abdominal obesity, was measured at the midpoint between the lower rib and iliac crest [47]. Additionally, BIA was employed to assess body composition in greater detail, providing estimates of fat mass and lean mass [44, 45]. Therefore, volunteers with a BMI ≥ 30 kg/m^2^ [46]; being females with WC values ≥ 105 cm [47] and body fat ≥ 35% [45], and males WC values ≥ 110 cm [47] and body fat ≥ 25% [45] were considered obese. Meanwhile, those with a BMI ≤ 26 kg/m^2^ [46]; and WC values ≤ 90 cm [47], and body fat < 35% [45] for females and ≤ 100 cm [47] and body fat < 25% [45] for males were considered normal weight controls. After selection, sociodemographic characteristics, such as time of edentulism, income, and educational level of volunteers were collected.

### 2.2 Periodontal Condition

Probing depth, clinical attachment loss, gingival recession, and bleeding on probing of abutment teeth were registered with a William's periodontal probe, at six sites per tooth (buccal, distobuccal, mesiobuccal, lingual, mesio‐lingual, disto‐lingual) [48]. To report the results for probing depth, clinical attachment loss, and gingival recession, the highest value among all six sites of each tooth was considered during data analysis. The mean and standard deviation were then calculated based on these values for each abutment tooth. Meanwhile, the visual plaque index of abutment teeth, as described by the modified O'Leary Index [48, 49] was recorded at the four main tooth surfaces: buccal, lingual, mesial, and distal, without the use of a disclosing agent. Visual plaque index and bleeding on probing values per abutment tooth were calculated according to the formula: number of surfaces with plaque/bleeding × 100/number of teeth × 4 [48, 49].

### 2.3 Maximum Bite Force

A force transducer (Spider 8; Hottinger Baldwin Messtechnik GmbH, Darmstadt, Germany) and two pressure sensors (FSR No. 151, 1.2 mm diameter, 5.6 mm thickness; Interlink Electronics Inc., Camarillo, CA, USA) [38] were used to measure MBF. All participants previously used unsatisfactory maxillary and mandibular dentures, which were in place during the pre‐rehabilitation MBF assessments. After oral rehabilitation, the evaluations were performed with the new maxillary CD and mandibular RPDs. Two sets of sensors, wrapped in plastic film to protect against moisture, were positioned bilaterally between the first artificial upper and lower molars [38]. Participants were then asked to occlude with maximum force on the set for 7 s [38]. A 5‐min rest was allowed, and the procedure was repeated [38]. The signals were recorded and analysed using the Catman Easy software (version 1.0, Hottinger Baldwin Messtechnik GmbH). The highest value obtained was considered the MBF, expressed in kilogram‐force (kgf) [38].

### 2.4 Oral Health‐Related Quality of Life

The Portuguese version of the OHIP‐14 questionnaire3 [9] was used to evaluate the OHRQoL. The analysis was performed at baseline (old prostheses) and after 6 and 12 months of new prostheses insertion. This questionnaire comprises 14 items assigned to seven domains: functional limitation, physical pain, psychological discomfort, physical disability, psychological disability, social disability, and handicap. Subjects were asked to rate the frequency in which they had experienced the impact of each OHIP‐14 item in the last 6 months on a five‐point Likert scale (never (0), rarely (1), sometimes (2), often (3), to almost always (4)). OHIP‐14 scores range from 0 to 56, with a higher score indicating a more negative impact of oral health conditions on an individual's quality of life [39].

### 2.5 Statistical Analysis

Results were analysed using the SPSS Statistics software (Version 25.0, IBM Corporation, Chicago, IL, USA) with a significance level of 5%. Data were first submitted to the Shapiro–Wilk test to verify the normality of distribution. Following this, Mauchly's sphericity test was applied. Once the prerequisites of normality and sphericity were met, the Analysis of Variance (ANOVA) for repeated measures was applied followed by the Bonferroni post hoc test for multiple comparisons between time points (baseline vs. after new prostheses use at different time points), and groups (obese vs. normal‐weight).

## 3 Results

**FIGURE 1:**
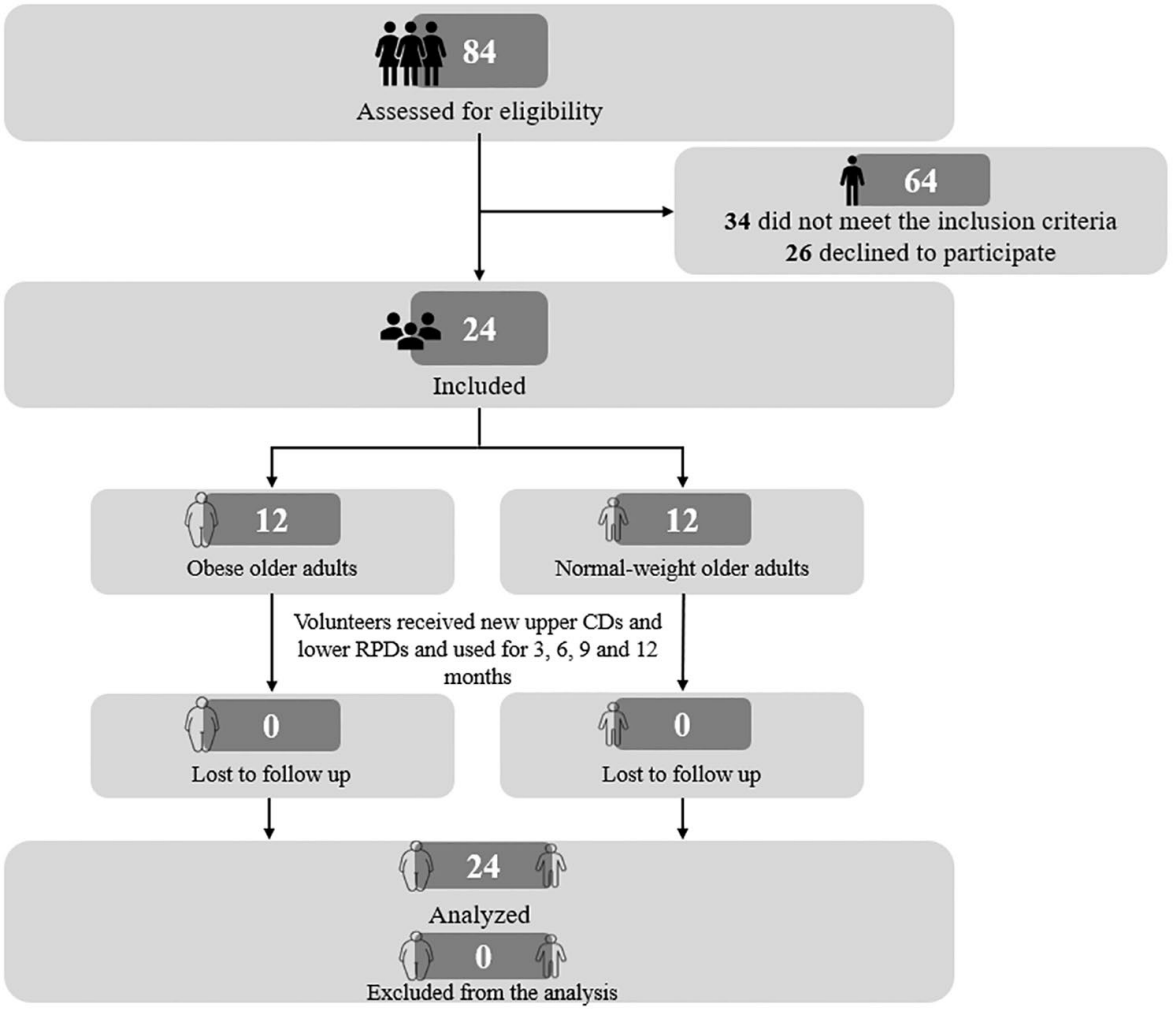
Flowchart of patient recruitment.

Eighty‐four volunteers were initially screened, and 34 were excluded for not meeting the eligibility criteria (22 with severe periodontal disease and advanced bone loss, and 12 with lower salivary flow rate) (Figure 1). Of the 50 eligible participants, 26 declined to participate. The final sample comprised 24 older adults, including 12 obese (6 women and 6 men) and 12 normal‐weight controls (6 women and 6 men), with a mean age of 70.90 ± 5.84 years. All obese participants and five controls had medically controlled arterial hypertension. Oral and sociodemographic characteristics are presented in Table 1.

**TABLE 1:** Mean values and standard deviation of oral and sociodemographic characteristics of the studied population.

Characteristics	Obese (*n* = 12)	Normal‐weight (*n* = 12)	*p* *
Age (years)	71.90 ± 5.00	69.90 ± 6.69	0.459
Time of edentulism (years)	32.60 ± 13.70	30.10 ± 11.11	0.659
Income (R$)	2990.00 ± 1767.26	2900.00 ± 1706.85	0.909
Educational level (years)	5.60 ± 3.37	6.40 ± 3.86	0.628
Height (cm)	161.20 ± 9.17	161.50 ± 5.42	0.930
Weight (kg)	86.11 ± 9.45	57.93 ± 7.86	< 0.001
Waist circunference (cm)	121.70 ± 10.90	69.20 ± 6.51	< 0.001
BMI (kg/m^2^)	33.26 ± 4.29	22.25 ± 3.06	< 0.001
Salivary flow rate	1.20 ± 0.42	1.31 ± 0.40	0.677

Abbreviations: cm, centimetres; kg, kilograms; m, meters; R$, BRL.

* Independent *t*‐test was performed, *p* < 0.05.

Table 2 presents the periodontal condition of the 48 abutment teeth evaluated. Obese individuals showed significantly higher bleeding on probing at all evaluation periods (*p* = 0.02). In the control group, bleeding on probing and visible plaque index decreased significantly after the insertion of the new prostheses and remained low throughout the study period (*p* = 0.01). In contrast, obese participants who initially exhibited higher plaque index levels with their old prostheses, improved this index following the new prostheses insertion (*p* = 0.03), with no significant changes thereafter (*p* > 0.05).

**TABLE 2:** Periodontal condition of RPD abutment teeth before and after new prostheses insertion in obese and normal‐weight older adults.

Groups	Prostheses insertion	Probing depth (mm)	Clinical attachment loss (mm)	Gingival recession (mm)	Bleending on probing (%)	Visible plaque index (%)
Normal‐weight	Before	2.00 ± 0.67Aa	3.20 ± 0.63Aa	1.20 ± 0.63Aa	43.75 ± 10.62Ba	52.50 ± 5.27Aa
	3 months	2.20 ± 0.42Aa	4.00 ± 0.82Aa	1.80 ± 0.63Aa	33.75 ± 11.85Bb	39.50 ± 9.04Ab
	6 months	2.20 ± 0.79Aa	4.20 ± 0.92Aa	2.00 ± 0.47Aa	28.75 ± 10.29Bb	32.25 ± 11.86Ab
	9 months	2.00 ± 0.47Aa	4.20 ± 0.63Aa	2.20 ± 0.42Aa	21.25 ± 10.29Bb	27.75 ± 5.74Ab
	12 months	1.60 ± 0.52Aa	3.10 ± 0.74Aa	2.30 ± 0.48Aa	18.75 ± 6.03Bb	22.25 ± 11.86Ab
Obese	Before	2.30 ± 0.68Aa	4.20 ± 0.79Aa	1.90 ± 1.10Aa	67.50 ± 14.67Aa	53.75 ± 6.04Aa
	3 months	2.70 ± 0.48Aa	5.00 ± 1.25Aa	2.30 ± 1.16Aa	56.25 ± 8.84Aa	39.00 ± 12.91Ab
	6 months	2.50 ± 0.71Aa	5.10 ± 1.29Aa	2.60 ± 1.08Aa	45.00 ± 8.74Aa	36.25 ± 8.43 Ab
	9 months	1.90 ± 0.32Aa	4.70 ± 1.06Aa	2.80 ± 0.92Aa	43.75 ± 8.84Aa	30.00 ± 8.74Ab
	12 months	2.20 ± 0.42Aa	4.40 ± 1.13Aa	2.40 ± 0.88Aa	41.25 ± 8.44Aa	25.50 ± 10.54Ab

*Note:* Data are presented as mean ± standard deviation. Distinct uppercase letters indicate differences between groups; distinct lowercase letters indicate differences between time points (*p* < 0.05, Repeated measures ANOVA).

Table 3 presents MBF results for both obese and normal‐weight groups, before and after insertion of the new prostheses across all follow‐up periods. Obese participants consistently showed higher MBF values compared to controls, both before (*p* = 0.002) and at all post‐insertion time points (3 months *p* = 0.002, 6 months *p* = 0.004, 6 months *p* = 0.006, 12 months *p* = 0.002). In addition, both groups showed a significant increase in MBF following the replacement of old prostheses (*p* = 0.005 for the experimental group; *p* < 0.001 for controls).

**TABLE 3:** Maximum bite force (kg/f) before and after prostheses insertion in obese and normal‐weight older adults.

Groups	Prostheses insertion	MBF
Normal‐weight	Before	12.21 ± 1.31Ba
	3 months	13.32 ± 1.26Bb
	6 months	13.47 ± 1.12Bb
	9 months	13.48 ± 1.12Bb
	12 months	13.51 ± 1.11Bb
Obese	Before	15.06 ± 0.84Aa
	3 months	16.12 ± 0.76Ab
	6 months	16.01 ± 0.80Ab
	9 months	15.94 ± 0.74Ab
	12 months	16.09 ± 0.74Ab

*Note:* Data are presented as mean ± standard deviation. Distinct uppercase letters indicate differences between groups; distinct lowercase letters indicate differences between time points (*p* < 0.05, Repeated measures ANOVA).

Abbreviation: MBF, maximum bite force.

Table 4 shows OHIP‐14 overall scores. The obese group reported significantly higher overall scores—indicating lower OHRQoL—both before and at 6 and 12 months after new prostheses insertion (*p* = 0.01). Despite this, both groups experienced a significant reduction in OHIP‐14 scores following the insertion of new prostheses (*p* = 0.02), with further improvement observed in the obese group at 12 months (*p* > 0.05). Regarding specific OHIP‐14 domains, obese participants scored higher in all domains except physical pain compared to controls after 6 months (*p* < 0.05). Within‐group comparisons showed that the obese group experienced significant improvements across most domains after 6 and 12 months, except for social disability (*p* = 0.002). In contrast, the controls showed improvements only in physical pain and psychological disability domains (*p* < 0.001).

**TABLE 4:** OHIP‐14 mean scores and standard deviation before and after new protheses insertion in obese and normal‐weight older adults.

Groups	Prostheses insertion	OHIP‐14 domains							
		Functional limitation	Physical pain	Psychological discomfort	Physical disability	Psychological disability	Social disability	Handicap	Overall
Normal‐weight	Before	1.80 ± 1.55Ba	4.20 ± 0.63Aa	2.20 ± 1.87Aa	2.90 ± 1.79Aa	2.40 ± 1.35Aa	0.90 ± 1.52 Aa	1.10 ± 1.37 Aa	16.00 ± 6.18 Ba
	6 months	0.70 ± 0.83Ba	1.50 ± 0.97Ab	0.40 ± 0.52Ba	0.70 ± 0.68Ba	0.30 ± 0.48Bb	0.20 ± 0.42 Ba	0.30 ± 0.48 Ba	4.10 ± 3.32 Bb
	12 months	0.30 ± 0.68Ba	1.60 ± 0.70Ab	0.30 ± 0.68Aa	0.70 ± 1.06Aa	0.10 ± 0.32Bb	0.10 ± 0.32 Aa	0.10 ± 0.32 Aa	3.10 ± 2.38 Bb
Obese	Before	4.80 ± 1.03Aa	5.20 ± 0.79Aa	4.00 ± 1.05Aa	5.50 ± 1.51Aa	3.70 ± 0.82Aa	1.70 ± 1.42 Aa	2.80 ± 0.92 Aa	27.70 ± 2.16 Aa
	6 months	2.30 ± 0.82Ab	2.60 ± 0.52Ab	1.60 ± 0.70Ab	1.90 ± 0.74Ab	1.10 ± 0.32Ab	0.90 ± 0.32 Aa	1.00 ± 0.47 Ab	11.40 ± 1.43 Ab
	12 months	1.50 ± 0.71Ab	1.70 ± 0.82Ab	1.30 ± 0.48Ab	1.10 ± 0.57Ab	0.80 ± 0.42Ab	0.70 ± 0.48 Aa	0.70 ± 0.48 Ab	7.80 ± 1.69 Ac

*Note:* Distinct uppercase letters indicate differences between groups; distinct lowercase letters indicate differences between time points (*p* < 0.05, Repeated measures ANOVA).

Abbreviation: OHIP‐14, Oral Health Impact Profile‐14.

## 4 Discussion

The findings of this study did not support the null hypothesis. Obese participants consistently exhibited higher bleeding on probing values and greater MBF than their normal‐weight counterparts across all evaluation periods. In contrast, they presented significantly lower OHRQoL scores, suggesting that obesity may negatively affect both oral health status and subjective well‐being. Nevertheless, oral rehabilitation with new removable prostheses led to marked improvements in MBF, OHIP‐14 scores, and plaque control in both groups. These results indicate that prosthetic treatment effectively enhanced masticatory performance and perceived oral health–related quality of life, regardless of obesity status.

The sociodemographic profile of older adult participants in this study was consistent with previous research [50, 51], characterised by low educational level [50] and a history of cardiovascular and hypertensive conditions [51]. Notably, all participants had normal salivary flow, despite the known association between polypharmacy and xerostomia or hyposalivation [52]. This inclusion criterion aimed to minimise bias in outcomes related to masticatory function, periodontal health, and OHRQoL.

Regarding the periodontal condition, comparisons between groups showed that obese participants demonstrated significantly higher bleeding on probing of the abutment teeth at all evaluation time points, aligning with prior studies [53, 54, 55] that reported greater gingival inflammation in individuals with higher BMI. Obesity is associated with elevated salivary proinflammatory cytokines [56, 57], which contribute to chronic systemic inflammation [58]. This inflammatory state impairs immune response [59], increasing susceptibility to periodontal infections. Additionally, the diet commonly associated with obesity—rich in processed and ultra‐processed foods—has been linked to higher rates of periodontitis [60, 61, 62]. Such diets promote the accumulation of inflammatory biomarkers (e.g., IL‐33, MCP‐1) in the gingival crevicular fluid [62], further increasing the risk of gingival inflammation in obese older adults.

Following the insertion of new prostheses, both groups exhibited a reduction in visual plaque index (Table 2). This contrasts with findings from do Amaral et al. [49] mainly due to differences in prosthesis design [46, 63]. According to the authors [47], the design of the retainers increases plaque accumulation and may cause deeper probing depth and higher gingival index. Correia et al. [63] also pointed out that occlusal clasps are associated with a worse periodontal condition when compared to gingival clasps, which may be explained by the larger contact area of the tooth with the occlusal part of the clasp, which may favour biofilm accumulation [63]. In the present study, only gingival clasps were used, reducing the contact area with abutment teeth and likely facilitating plaque control. Furthermore, proper RPD design, including rigid components, well‐planned frameworks, and adequate acrylic base extension, contributes to a more even distribution of occlusal forces and supports periodontal stability [64, 65, 66].

All participants followed a strict maintenance protocol with 3‐month recall visits, which likely contributed to the stable periodontal parameters observed (Table 2). These findings are consistent with prior studies [30, 67, 68, 69, 70, 71] which emphasise the importance of oral hygiene instruction and regular recall appointments for long‐term abutment tooth survival. Tada et al. [68] specifically reported that semiannual periodontal recalls helped prevent tooth loss in RPD wearers. Considering that obese participants showed consistently higher bleeding on probing, a more frequent re‐assessment—every 3 months—is recommended to reinforce oral hygiene practices and prevent the progression of periodontal disease in this population.

Unexpectedly, the obese group exhibited higher MBF values, despite consuming a diet rich in ultra‐processed foods, which typically requires less masticatory effort and might even be expected to reduce bite force [72]. This counterintuitive finding may be related to a metabolic profile commonly observed in obesity, often characterised by hyperinsulinemia. Insulin exerts an anabolic effect that facilitates nutrient uptake by tissues, including muscle, thereby supporting protein synthesis and muscle growth [73, 74]. Such a metabolic environment promotes the development and maintenance of muscle mass [74], which may contribute to the elevated MBF in obese individuals. Therefore, obesity‐related hormonal alterations likely modulate masticatory muscle strength.

Considering the comparisons before and after the insertion of the new prostheses, MBF increased in both groups after 3 months, and remained stable through 6, 9, and 12 months (Table 3). This aligns with evidence that RPDs restore posterior occlusal contacts, thereby improving masticatory function [35]. The mechanical properties of cobalt‐chromium alloy used in the RPD frameworks—particularly its rigidity and durability—contribute to prosthesis stability and retention [35]. Structural components such as clasps and rests help evenly distribute masticatory forces across the dental arch [75], supporting consistent occlusal performance [35]. Furthermore, proper occlusal arrangement and adjustment enhance MBF and prosthetic stability [76] by ensuring balanced contact between artificial and natural teeth, minimizing abutment overload [76, 77]. Occlusal adjustments also eliminate premature contacts, facilitating neuromuscular adaptation and maintaining MBF over time [78, 79, 80]. Correct posterior tooth positioning further promotes even force distribution, supporting long‐term prosthetic function [81]. Together, these factors are critical for the clinical success and longevity of RPDs and play a key role in improving patient comfort and OHRQoL [82].

In terms of OHRQoL, the OHIP‐14 scores revealed consistently lower values in obese participants compared to normal‐weight controls at all evaluation periods (Table 4), indicating a significant negative influence of obesity on OHRQoL. These findings align with previous studies [83, 84], showing that individuals with nutritional disorders, particularly obesity, are up to three times more likely to report impaired OHRQoL [83, 84]. This negative impact may be partially attributed to poorer general health, socioeconomic disparities, and reduced autonomy over food choices [85], which are common in older adults and can influence dietary patterns. Furthermore, several studies [86, 87, 88] have reported taste perception deficits in obese individuals, leading to a preference for sweet or carbohydrate‐rich foods [88], which may worsen oral health problems and further reduce OHRQoL.

Despite these challenges, both groups experienced significant improvements in OHRQoL following the insertion of new prostheses. Oral rehabilitation plays a critical role in restoring quality of life in older adults [89]. While Choong et al. [90] revealed that RPDs improve OHRQoL in the short term (up to 6 months), long‐term effects beyond this period remained unclear. In contrast, our results showed sustained OHRQoL improvements over 12 months, underscoring the functional and psychosocial benefits of well‐fitted RPDs.

It is important to highlight the limitations of the present study. Different aspects of the masticatory function were not assessed including muscle electromyography activity, masticatory muscle movements, and thickness of masticatory muscles, limiting assumptions related to masticatory muscle performance. In addition, dietary intake and nutritional status were not evaluated, which may act as confounding factors influencing periodontal condition. Nonetheless, the study is strengthened by its comprehensive approach to assessing obesity. In addition to BMI, it employed BIA and waist circumference, offering a more accurate picture of body composition in older adults [43]. BIA is especially valuable in elderly populations, where BMI alone may underestimate fat mass, while waist circumference provides a robust indicator of central adiposity, closely linked to cardiometabolic risk [45]. Together, these measures offer a comprehensive assessment of obesity, considering not only total body weight but also fat distribution and overall body composition [42, 43, 45], thereby enhancing the robustness and relevance of the study findings. Nevertheless, future studies including additional masticatory analyses and dietary assessments are needed to explore the potential influence of these parameters on periodontal health in obese and normal‐weight older adults. Moreover, comparisons between conventional and implant‐supported prostheses may offer further insight into how different rehabilitation strategies impact oral health outcomes in the context of obesity and aging.

## 5 Conclusion

The mid‐term use of removable prostheses did not negatively impact the periodontal health of either obese or normal‐weight older adults. However, obese individuals consistently exhibited higher bleeding on probing of abutment teeth. Both groups initially showed high plaque levels with their old prostheses, which significantly decreased after the insertion of new RPDs and remained stable thereafter.

Obese individuals also demonstrated higher MBF than normal‐weight controls, with both groups showing further MBF improvements 3 months post‐rehabilitation. Despite these functional gains, OHRQoL remained lower in the obese group throughout the study.

Overall, the removable prostheses proved to be a safe and conservative treatment option for restoring edentulous areas in obese and normal‐weight older adults. However, maintaining periodontal health in the obese population requires reinforced oral hygiene education and adherence to regular follow‐up care.

## Author Contributions

G.F.F. and L.T.G. contributed to the acquisition of data and performed data analysis. T.M.C. and R.C.M.R.G. contributed to the conception and design of the study. G.F.F. and L.T.G. drafted the first version of the manuscript. T.M.S.V.G. and R.C.M.R.G. revised the manuscript critically. All authors read and approved the final version of the manuscript.

## Funding

This work was supported by Coordenação de Aperfeiçoamento de Pessoal de Nível Superior, Finance code 001.

## Conflicts of Interest

The authors declare no conflicts of interest.

## Data Availability

The data that support the findings of this study are openly available in Repositório de Dados de Pesquisa da Unicamp—REDU at https://redu.unicamp.br/, reference number https://doi.org/10.25824/redu/J8X8XT.
